# The geographic alignment of primary care Health Professional Shortage Areas with markers for social determinants of health

**DOI:** 10.1371/journal.pone.0231443

**Published:** 2020-04-24

**Authors:** Robin A. Streeter, John E. Snyder, Hayden Kepley, Anne L. Stahl, Tiandong Li, Michelle M. Washko

**Affiliations:** 1 National Center for Health Workforce Analysis (NCHWA), Bureau of Health Workforce (BHW), Health Resources and Services Administration (HRSA), U.S. Department of Health and Human Services (HHS), Rockville, Maryland, United States of America; 2 Office of Planning, Analysis, and Evaluation (OPAE), Health Resources and Services Administration (HRSA), U.S. Department of Health and Human Services (HHS), Rockville, Maryland, United States of America; The University of the South Pacific, FIJI

## Abstract

**Background:**

The Health Resources and Services Administration (HRSA), an agency within the U.S. Department of Health and Human Services (HHS), works to ensure accessible, quality, health care for the nation’s underserved populations, especially those who are medically, economically, or geographically vulnerable. HRSA-designated primary care Health Professional Shortage Areas (pcHPSAs) provide a vital measure by which to identify underserved populations and prioritize locations and populations lacking access to adequate primary and preventive health care–the foundation for advancing health equity and maintaining health and wellness for individuals and populations. However, access to care is a complex, multifactorial issue that involves more than just the number of health care providers available, and pcHPSAs alone cannot fully characterize the distribution of medically, economically, and geographically vulnerable populations.

**Methods and findings:**

In this county-level analysis, we used descriptive statistics and multiple correspondence analysis to assess how HRSA’s pcHPSA designations align geographically with other established markers of medical, economic, and geographic vulnerability. Reflecting recognized social determinants of health (SDOH), markers included demographic characteristics, race and ethnicity, rates of low birth weight births, median household income, poverty, educational attainment, and rurality. Nationally, 96 percent of U.S. counties were either classified as whole county or partial county pcHPSAs or had one or more established markers of medical, economic, or geographic vulnerability in 2017, suggesting that at-risk populations were nearly ubiquitous throughout the nation. Primary care HPSA counties in HHS Regions 4 and 6 (largely lying within the southeastern and south central United States) had the most pervasive and complex patterns in population risk.

**Conclusion:**

HHS Regions displayed unique signatures with respect to SDOH markers. Descriptive and analytic findings from our work may help inform health workforce and health care planning at all levels, and, by illustrating both the complexity of and differences in county-level population characteristics in pcHPSA counties, our findings may have relevance for strengthening the delivery of primary care and addressing social determinants of health in areas beset by provider shortages.

## Introduction

Health equity has been a key goal of Healthy People 2020, and the American Medical Association recently concluded work on a task force affirming the organization’s commitment to promote equity in care and improve health care access for all. [1, 2] Primary care has been recognized as a key means by which to promote health equity and care access–given its broad interaction with the population, its role as the chief point of direct patient contact within the greater health care system for many people, its degree of care continuity and function in comprehensive care coordination, and its typically strong level of appreciation for the social contexts in which patients live. [3] Indeed, primary care has been described as the bedrock upon which health care systems can achieve better individual and population health, access to health services, cost control, and experience and quality of care–and, as such, primary care has been the driving force behind many policy efforts to overcome health care barriers for disadvantaged populations in particular. [4]

Access to primary care, however, remains a significant challenge in the United States, as more than 6,000 areas in the nation have been classified as primary care Health Professional Shortage Areas (pcHPSAs), and several thousand areas and populations have also been classified as medically underserved. [5–7] The National Center for Health Workforce Analysis, an agency component within the federal Health Resources and Services Administration (HRSA) reports that by 2025, the U.S. will be short over 20,000 primary care providers, with particular workforce disparities in rural and underserved areas. [8] The Association of American Medical Colleges reports similar findings. [9] Beyond shortages, primary care providers and health care facilities in underserved areas face unique challenges, especially with provider recruitment and retention. [10] Yet, extensive prior research has indicated that a higher per-population density of primary care physicians is a strong predictor of better health outcomes across a number of domains. [11–15]

Well-recognized health disparities, including life expectancy, health-related quality of life, and disease severity, exist between individuals living in underserved areas and those living in metropolitan areas. [16–18] Social determinants of health (SDOH), including demographic, economic and social factors, can help explain these health disparities. [19–21] Socioeconomic status, race/ethnicity, and poverty are often among the strongest predictors of health, and households in rural areas experience significantly higher rates of poverty when compared with their urban counterparts. [22–25] Thus, the availability of health care professionals in underserved areas becomes a necessary but insufficient goal to achieving health equity, reducing disparities, and addressing social determinants of health. In line with this, the American College of Physicians, the nation’s largest medical specialty organization representing internal medicine physicians, recently published a call to action for promoting health equity, addressing social determinants of health, and reducing barriers to care by correcting primary care workforce shortages and through other means. [26]

HRSA, an agency within the U.S. Department of Health and Human Services (HHS), seeks to ensure accessible, quality health care to the nation’s underserved populations, especially those populations who are medically, economically, or geographically vulnerable. [27] HRSA accomplishes its mission by providing primary care to those in need through its extensive Health Center Program, as well as through programs to assist individuals living with HIV/AIDS, pregnant patients, mothers, children, and other populations who may lack adequate access to health care, whether due to provider shortages or other factors. [27–30] HRSA also seeks to strengthen and sustain health care delivery through programs that support the education, training, recruitment, and retention of health professionals and paraprofessionals. Collectively, HRSA provides funding through more than 90 health care and health services programs to over 3,000 grantees. [27]

To help target its health workforce-building programs to areas of need, HRSA works closely with State Primary Care Offices to designate Health Professional Shortage Areas (HPSAs), of which pcHPSAs are a subset. [31] Primary care HPSA designations are based in regulation and are scored on four broad measures of need: (i) Population-to-provider ratio; (ii) Percent of population below 100 percent of the Federal Poverty Level (FPL); (iii) Infant Health Index (based on the infant mortality rate or low birth weight rate); and (iv) Travel time to the nearest source of care (NSC). Primary care HPSAs may be geographically based, population based, or facility based. Geographic and population-based HPSAs may include whole counties or partial counties, with the provision that the defined shortage areas reflect rational areas for delivery of health care services. Facility-based HPSAs, as the name implies, identify facilities (e.g., correctional facilities, Indian Health Facilities, Federally Qualified Health Centers, etc.) that lack access to an adequate supply of health professionals. [31, 32]

HRSA-designated pcHPSAs have been found to be associated with areas of higher mortality and morbidity [33–35], and so can provide a useful measure by which to identify locations and populations lacking access to adequate primary and preventive health care. However, as a stand-alone measure, pcHPSAs cannot fully characterize the distribution of medically, economically, and geographically vulnerable populations because access to care is a complex, multifactorial issue that involves more than just the number of health care providers available. Factors such as race and ethnicity, poverty, education, housing, transportation, employment, and the system of justice–again, often characterized together by the term “social determinants of health”–all can reflect underlying social and economic patterns that may reduce access to health care services, and all have been found to be associated with poorer population health and higher prevalence of disease and injury in communities. [36] Hence, a more comprehensive picture of access to care can be discerned by overlaying HRSA’s pcHPSA categorizations with other markers of medical, economic, and geographic vulnerability. Such analyses have potential to help elucidate regional similarities and variations and to identify population complexities that may not be apparent when looking solely at pcHPSAs. More importantly, such analyses may help to guide health care planning and health workforce development initiatives, including training, recruitment, and retention efforts, in order to most effectively address the complex needs of vulnerable and at-risk populations.

The purpose of this study is to offer a county-level view of at-risk, high-need populations across the United States by examining pcHPSA designations for all three types of pcHPSAs (i.e., geographic, population-based, and facility-based pcHPSAs) together with other established markers of medical, economic, and geographic vulnerability. Findings from these analyses aim to guide enhanced health program resource planning, to the extent allowable by federal program statute, by providing a better understanding of locations and populations at risk for inadequate access to health care. These findings may be particularly relevant to strengthening the workforce charged with delivering primary care to highly vulnerable populations with complex needs.

## Methods

We first compiled information for all designated pcHPSAs using data from HRSA’s public-facing data portal (accessible via https://data.HRSA.gov). [6, 37] We then aggregated these data to derive counts of pcHPSAs by U.S. county or county equivalent using U.S. Census Bureau codes and 2017 geographic classifications. [38–42] Each 5-digit Census code is based on the former Federal Information Processing Standard (or FIPS) code and reflects a 2-digit state (or state equivalent) code and a 3-digit county (or county equivalent) code, thereby unambiguously capturing county-equivalent geographies including Alaska’s boroughs and census areas, the District of Columbia, Louisiana’s parishes, and Virginia’s independent cities. [42] For simplicity, the term “county” is used throughout this paper, and is consistent with the county-equivalent entities determined by the Census Bureau.

Counts of pcHPSAs reflected all pcHPSAs in each U.S. county that were designated as of July 1, 2017, and excluded pcHPSAs denoted as “proposed for withdrawal” or “withdrawn” as of this July 1, 2017 date, as well any pcHPSA that was designated after this date. We then linked the county-level counts to other county-level markers of medical, economic, and geographic vulnerability available from HRSA’s Area Health Resources File (AHRF). [42, 43] The AHRF is updated annually to reflect a wide range of population and health measures, including population estimates and demographic data from the Census Bureau, economic data from the Bureau of Labor Statistics, natality data from the National Center for Health Statistics (NCHS), and geographic designations from the U.S. Department of Agriculture (USDA).

Together, the selected markers examine 15 characteristics that have been established by the World Health Organization (WHO) [19, 20] and Healthy People 2020 [1] as indicators of population risk and inequities in medical, economic, and geographic domains for which county-level data were readily available for analysis (Table 1).

**Table 1:** Markers for medical, economic, and geographic vulnerability.

SDOH Marker	Year	Source	Condition for setting an SDOH marker equal to 1
(1) Older population	2017	AHRF	County estimate of proportion of population 65 and older exceeds the 75^th^ percentile of all US county-level estimates
(2) Low birth weight	2017	AHRF	County estimate of low birth weight births (per 1,000 live births) exceeds the 75^th^ percentile of all US county-level estimates
(3) Low income	2017	AHRF	County estimate of median household income is less than the 25^th^ percentile of all US county-level estimates
(4) Unemployment	2017	AHRF	County estimate of unemployment rate exceeds the 75^th^ percentile of all US county-level estimates
(5) Poverty	2017	AHRF	County estimate of proportion of population below the FPL exceeds the 75^th^ percentile of all US county-level estimates
(6) Deep poverty among adults 65 and older	2017	AHRF	County estimate of proportion of adults 65 and older in deep poverty exceeds the 75^th^ percentile of all US county-level estimates
(7) Deep poverty among children under 18	2017	AHRF	County estimate of proportion of children under 18 in deep poverty exceeds the 75^th^ percentile of all US county-level estimates
(8) Persistent poverty	1989 1999 2017	CRS	County is identified as a persistent poverty county by CRS, based on 1989, 1999, and 2017 poverty estimates
(9) Education	2017	AHRF	County estimate of proportion of adults 25 and older without a high school diploma exceeds the 75^th^ percentile of all US county-level estimates
(10) Race/ethnicity	2017	AHRF	County estimate of proportion of total non-White or Hispanic Origin individuals exceeds the 75^th^ percentile of all US county-level estimates
(11) Insurance	2017	AHRF	County estimate of the proportion of adults 64 and younger who lack health insurance and who are below 200 percent of the FPL exceeds the 75^th^ percentile of all US county-level estimates
(12) Sparse population (low population density)	2017	AHRF	Number of people per square mile of land area is less than the 25^th^ percentile of all US county-level estimates
(13) Rurality	2013	AHRF	County has a Rural-Urban Continuum Code of 7, 8, or 9 or an Urban Influence Code of 9 through 12
(14) Primary care HPSA county (pcHPSA county)	2017	AHRF, HPSA data file	County is categorized as a whole or partial county primary care HPSA in AHRF, or county has one or more pcHPSAs listed in the HPSA data file
(15) pcHPSA count (as of July 1, 2017)	2017	HPSA data file	Number of pcHPSAs in county exceeds the 75^th^ percentile of all US county-level pcHPSA counts

AHRF: Area Health Resources File, 2018–2019 Release. Available from: https://data.hrsa.gov/.

CRS: Congressional Research Service, The 10-20-30 Provision: Defining Persistent Poverty Counties.

Available from: https://fas.org/sgp/crs/misc/R45100.pdf.

HPSA: Health Professional Shortage Area. HPSA data file available from: https://data.hrsa.gov.

We recognize that many of these measures are inherently inter-related. For example, low education levels have a well-documented association with poverty. [44] Similarly, rates of low birth weight births, poverty levels, and population distribution are all elements that may be considered in the shortage designation scoring process for certain types of pcHPSAs [31, 32], and these elements may, in turn contribute to correlation among markers. However, several factors lead to the complementarity, rather than the redundancy, of our selected markers. These factors include the use of a consistent unit for analysis (i.e., the county), a consistent point in time (i.e., 2017 for the majority of markers), and a consistent benchmark (e.g., the upper 75^th^ percentile of corresponding national estimates for the majority of markers). In addition, with respect to the overlap of these markers with pcHPSA designation criteria, we would note that the majority of pcHPSAs do not directly incorporate county-wide estimates of low birth weight births, poverty levels, or population as criteria for designation. [32] Moreover, pcHPSA designations do not reflect comparisons with the upper quartiles of national estimates. And finally, among those pcHPSAs that are geographic, most do not include an entire county. For these reasons, we suggest that, rather than “double-counting” certain public health measures, the county-level markers used in our analyses can collectively help to reveal regional patterns in population risk and domain complexity.

With two exceptions, all characteristics reflect 2017 data, the latest year for which the majority of data classifiers are available. Exceptions include CRS’ designation of persistent poverty counties, which utilizes county poverty rates in 1989, 1999, as well as 2017 [45], and rurality codes, which utilize 2013 codes developed by the USDA’s Economic Research Service (ERS). [46]

Each county was assigned a score of either 0 or 1 for each of these 15 markers, using the decision rules or conditions listed in Table 1. As shown, score assignments were generally based on comparisons to national levels, and a score of 1 typically indicates that a county lies in the highest quartile nation-wide for a given marker. For example, the economic marker for unemployment was assigned a score of 1 if the county-level unemployment rate was higher than the 75^th^ percentile of unemployment rates across all U.S. counties in 2017.

For three markers (i.e., the persistent poverty marker (Marker 8), the rurality marker (Marker 13), and the pcHPSA county marker (Marker 14)), scoring was performed using a pre-defined classification scheme rather than by comparison to national quartiles. An example of this approach may be seen in the scoring for the rurality marker. Here, a score of 1 was assigned if ERS classifies the county with either of the following: (i) a 2013 Rural/Urban Continuum Code (RUCC) of 7, 8, or 9 [47]; or (ii) a 2013 Urban Influence Code (UIC) of 9, 10, 11, or 12 [48]. RUCC codes of 7, 8, and 9 correspond to the following 3 categories respectively: (i) County has an urban population of 2,500 to 19,999, and the county is not adjacent to a metro area; (ii) County is either completely rural or the county has an urban population of less than 2,500, and the county is adjacent to a metro area; (iii) County is either completely rural or the county has an urban population of less than 2,500, and the County is not adjacent to a metro area. UIC codes 9 through 12 correspond to the following 4 categories respectively: (i) County is a noncore county adjacent to a micro area, and the county contains a town of at least 2,500 residents; (ii) County is a noncore county adjacent to a micro area, and the county does not contain a town of at least 2,500 residents; (iii) County is a noncore county, is not adjacent to a metro or micro area, and contains a town of at least 2,500 residents; (iv) County is a noncore county, is not adjacent to a metro or micro area, and does not contain a town of at least 2,500 residents. The use of UIC codes to identify noncore counties as rural aligns with the most rural designation in the urban/rural continuum developed by the National Center for Health Statistics [49], a classification that has been shown to capture differences in how health access measures and outcomes vary across urban and rural geographies. [25] Augmenting the UIC codes with RUCC codes helps provide more nuanced insight into potential geographic disparities related to access to and availability of health care, including the travel time that may be required for a county resident to visit a health care provider.

After the marker scoring process was completed, we examined how SDOH markers were distributed across the United States as well as how they aligned with HRSA-designated pcHPSAs, and we explored how marker frequencies differed by HHS Region [https://www.hhs.gov/about/agencies/iea/regional-offices/index.html]. HHS Regions work closely with state, local, and tribal jurisdictions to address the health and human services needs of communities and individuals served by HHS programs and policies [50], and thus, HHS Regions offer a useful lens through which to consider population risk characteristics. We supported our analyses with selected maps to elucidate regional differences in marker totals.

An important counterpoint to looking at marker frequencies and co-occurring markers is understanding regional variation and disparity in population risk characteristics. Using Multiple Correspondence Analysis (MCA) [51–53], a nonparametric method to explore patterns and variability in categorical variables, we identified the predominant marker contributions to overall variability in county-level SDOH markers across pcHPSA counties in each HHS Region. Due to the relatively high proportion of missing values (25 percent), the low birth weight rate marker was excluded from the MCA analyses.

All analyses were conducted using R, Version 3.5.1 (R Foundation for Statistical Computing, Vienna, Austria), and SAS, Version 9.4 (SAS Institute, Inc., Cary, NC). Maps were prepared using R’s open source package, usmaps, developed by Paolo Di Lorenzo under a GNU General Public License (GPL), Version 3 (https://cran.r-project.org/package=usmap).

## Results

### National overview

Across the 50 U.S. States and the District of Columbia, there were 3,142 counties and county-equivalent jurisdictions in 2017, and only 131 (4.2 percent) had no markers of population risk in our analysis, with the remaining, vast majority of counties having between 1 and 13 markers. This suggests that nearly all U.S. counties have segments of their populations with one or more risk factors for health service access limitations and poor population health. Moreover, there were 206 U.S. counties (6.6 percent) with 10 or more markers, and 9 counties (0.3 percent) had the maximum, 13 markers, suggesting higher overall risk levels for populations within those counties. These 9 counties lay entirely within either HHS Region 4 (Alabama, 2 counties; Georgia, 1 county; Mississippi, 4 counties) or Region 6 (New Mexico, 1 county; Texas, 1 county), roughly the southeastern and south central United States, respectively.

### Primary care HPSA counties

Looking specifically at pcHPSA counties, 89 percent of U.S. counties were classified as pcHPSA counties in our analysis (Table 2), as determined through a regulatory process using health care provider and delivery metrics. This proportion may appear high but likely reflects the recognized national-level deficit in primary care providers. [8, 9] The proportion varied by HHS Region, with the highest percentages of pcHPSA counties found in Region 9 (100 percent) and Region 10 (98 percent), which encompass the nation’s western-most states. In four additional Regions across the nation (Region 1, Region 4, Region 6, and Region 8), more than 90 percent of all counties were classified as pcHPSA counties. The lowest proportion of counties classified as pcHPSA counties was found in Region 3, encompassing the Mid-Atlantic states, where 79 percent of counties were considered pcHPSA counties.

**Table 2:** Summary of pcHPSA counties, by HHS Region, 2017.

HHS Region	US States + DC	Total Number of Counties	Total Number of pcHPSA-Counties	pcHPSA Counties, as Percentage of Total Number of Counties	pcHPSA Counties with > 6 Markers	pcHPSA Counties with > 6 Markers, as Percentage of Total Number of Counties
1	CT, ME, MA, NH, RI, VT	67	62	92.5%	[none]	—
2	NJ, NY	83	69	83.1%	3	3.6%
3	DC, DE, MD, PA, VA, WV	283	223	78.8%	49	17.3%
4	AL, FL, GA, KY, MS, NC, SC, TN	736	672	91.3%	288	39.1%
5	IL, IN, MI, MN, OH, WI	524	443	84.5%	22	4.2%
6	AR, LA, NM, OK, TX	503	459	91.3%	184	36.6%
7	IA, KS, MO, NE	412	358	86.9%	29	7.0%
8	CO, MT, ND, SD, UT, WY	291	271	93.1%	44	15.1%
9	AZ, CA, HI, NV	95	95	100%	24	25.3%
10	AK, ID, OR, WA	148	145	98.0%	19	12.8%
**All Regions**	**50 US States + DC**	**3,142**	**2,797**	**89.0%**	**662**	**21.1%**

Total counties includes county-equivalent jurisdictions (e.g., parishes in Louisiana).

The total number of counties and county equivalents reflects the number of jurisdictions in 2017.

pcHPSA: HRSA-designated primary care Health Professional Shortage Area.

**Fig 1:**
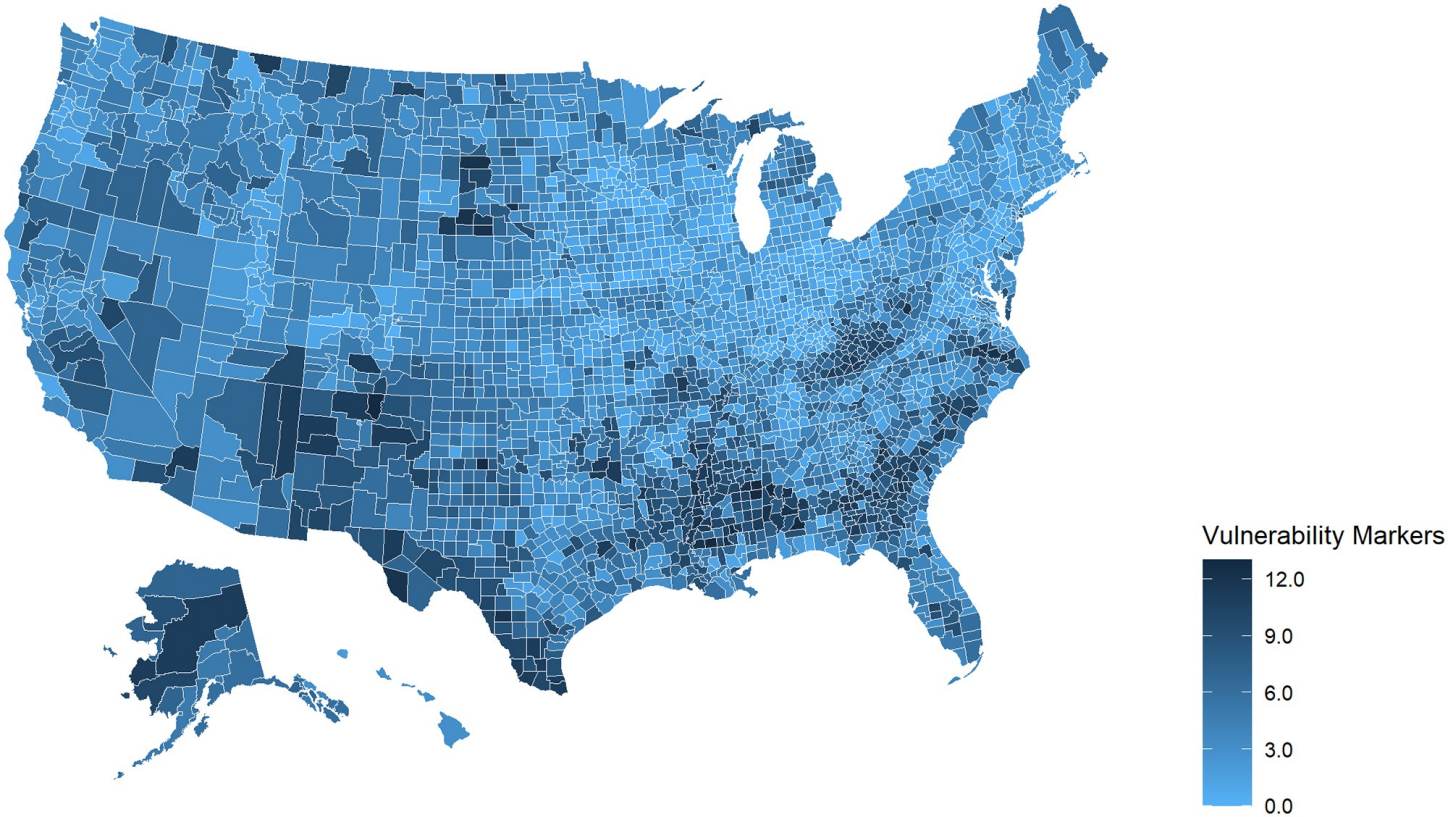
Markers of medical, economic, and geographic vulnerability in the United States, 2017. Map prepared using R’s open source package, usmaps, developed by Paolo Di Lorenzo under a GNU General Public License (GPL), Version 3 (https://cran.r-project.org/package=usmap).

**Fig 2:**
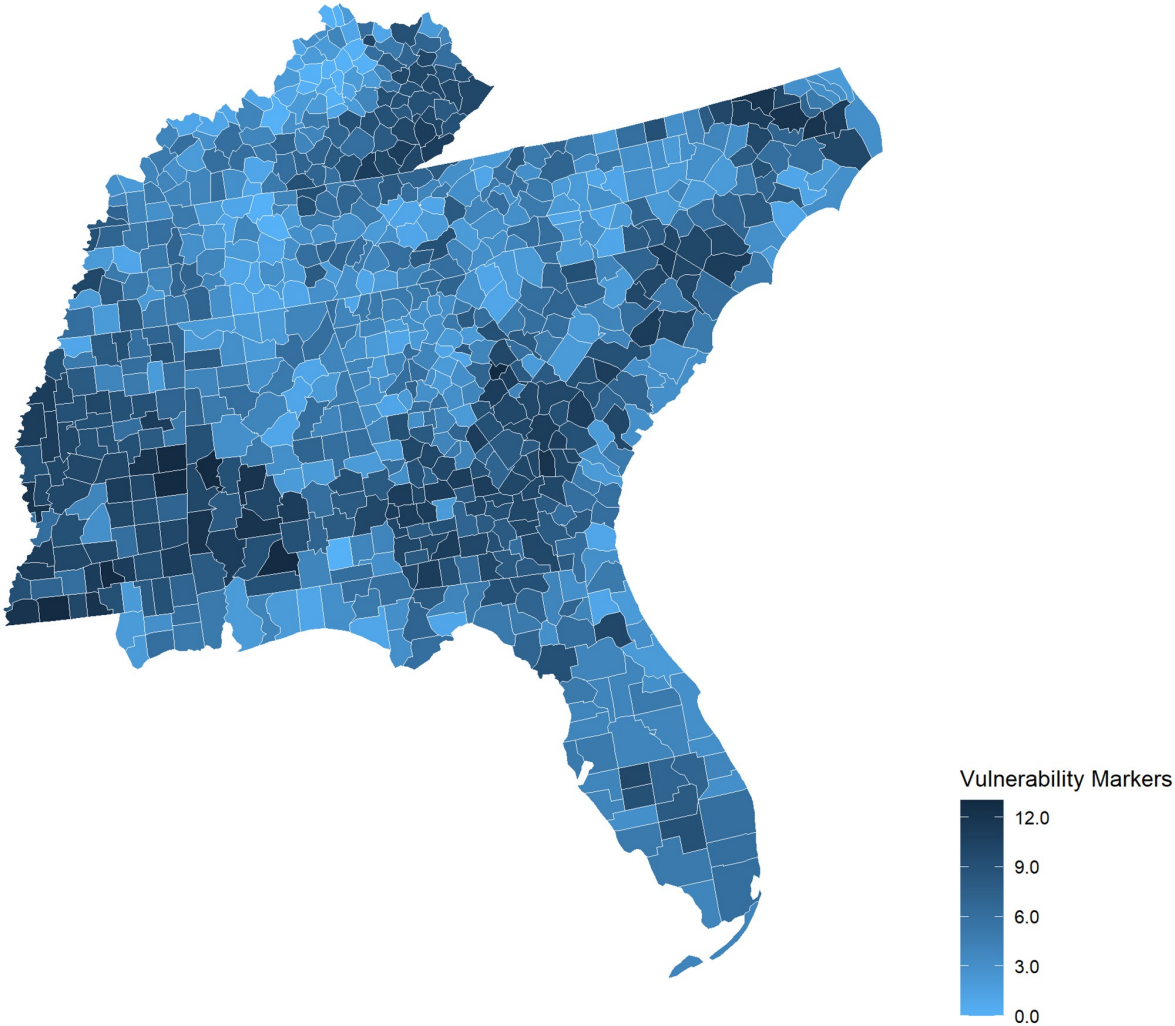
Markers of medical, economic, and geographic vulnerability, HHS Region 4, 2017 Alabama, Florida, George, Kentucky, Mississippi, North Carolina, South Carolina, Tennessee. Map prepared using R’s open source package, usmaps, developed by Paolo Di Lorenzo under a GNU General Public License (GPL), Version 3 (https://cran.r-project.org/package=usmap).

**Fig 3:**
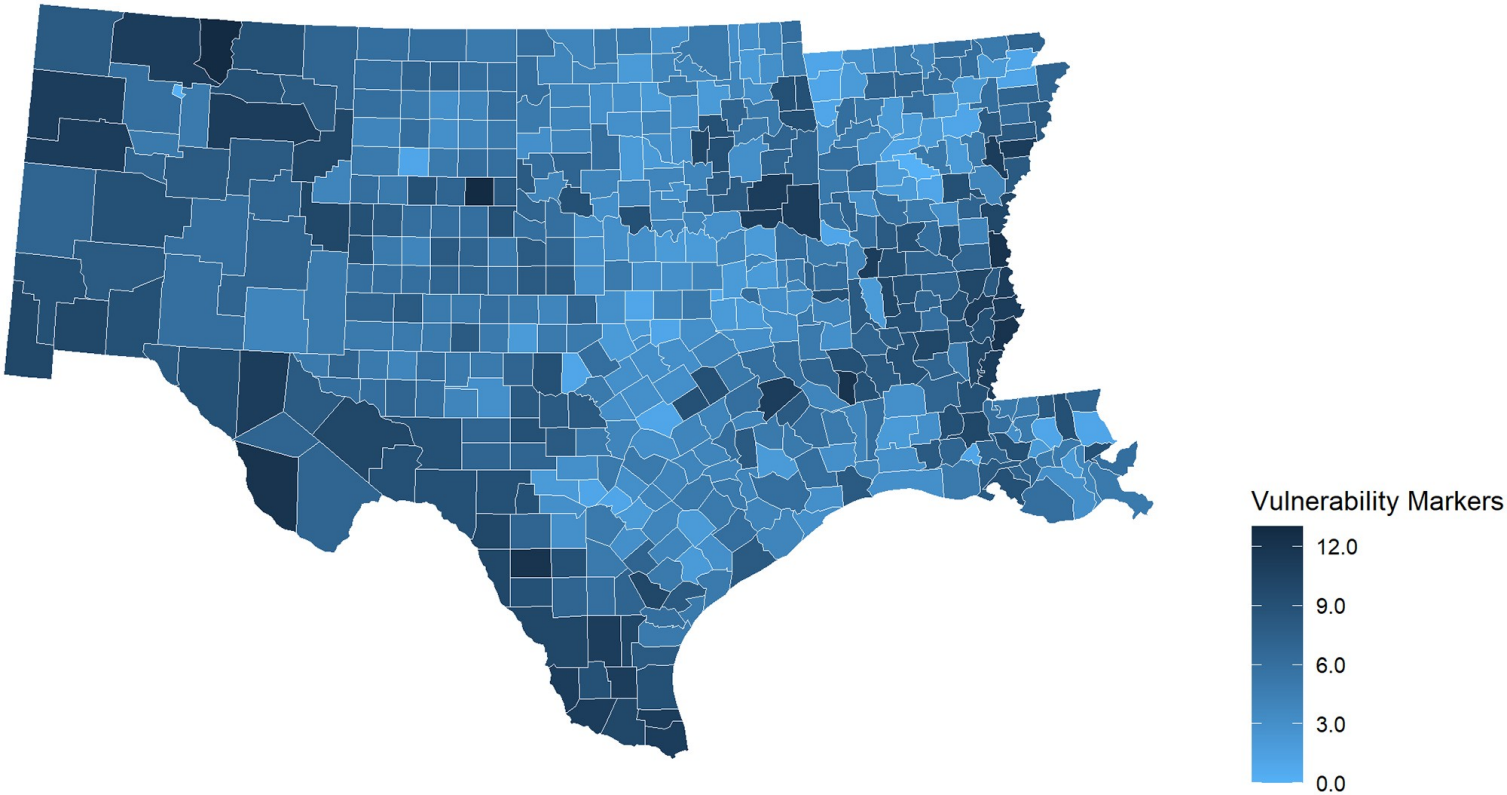
Markers of medical, economic, and geographic vulnerability, HHS Region 6, 2017 Arkansas, Louisiana, New Mexico, Oklahoma, Texas. Map prepared using R’s open source package, usmaps, developed by Paolo Di Lorenzo under a GNU General Public License (GPL), Version 3 (https://cran.r-project.org/package=usmap).

The picture becomes more complicated when we looked by HHS Region at the number of pcHPSA counties with more than six SDOH markers. This threshold is based on the 75^th^ percentile of the total number of markers across all U.S. counties, and reveals marked differences. Disparities were particularly evident in HHS Region 4 and Region 6, which include states in the southeastern and south central United States and where more than a third of the counties in each Region were pcHPSA counties with more than 6 of the 15 studied SDOH markers. Figs 1 and 2 and 3 illustrate the county-level variability in the number of markers, focusing first across all states, and then viewing HHS Region 4 and Region 6 more closely.

### SDOH marker co-occurrence and variability

Looking at the most common markers across pcHPSA counties by HHS Region, we see that each HHS Region reveals a signature (Table 3). For example, in HHS Regions 1, 2, and 3, one of the most frequent SDOH markers was the one for the number of pcHPSAs within the county (Marker 15, Table 1), with frequencies of 73 percent, 51 percent, and 33 percent, respectively. In Regions 1 and 3, having a high proportion of adults 65 and older was a marker in 32 percent and 37 percent of pcHPSA counties, respectively, and in Region 1, rurality was a common marker (26 percent of pcHPSA counties). Education was a dominant marker in Regions 4 and 6 (54 percent and 58 percent, respectively), while race/ethnicity was among the three most frequent markers in Regions 2, 6, and 9 (26 percent, 54 percent, and 59 percent, respectively). Unemployment was a consistent marker in Regions 5, 9, and 10 (25 percent, 41 percent, and 42 percent). In Regions 7 and 8, rurality (52 percent and 71 percent, respectively) together with sparse population (43 percent and 80 percent, respectively) were among the most frequent markers. Across all U.S. pcHPSA counties, the most common markers were rurality (36 percent of pcHPSA counties) and having multiple pcHPSAs in a county (30 percent of pcHPSA counties). Markers for education, low income, poverty, and sparse population were each present in 27 percent of pcHPSA counties nationwide. Marker frequencies for all markers and HHS Regions are provided in the S1 File.

**Table 3:** Predominant SDOH markers in pcHPSA counties, by HHS Region, 2017.

HHS Region	US States + DC	Most Frequent Marker	Second Most Frequent Marker	Third Most Frequent Marker
1	CT, ME, MA, NH, RI, VT	pcHPSA count (73%)	Older population (32%)	Rurality (26%)
2	NJ, NY	pcHPSA count (51%)	Unemployment (41%)	Race/ethnicity (26%)
3	DC, DE, MD, PA, VA, WV	Unemployment (38%)	Older population (37%)	pcHPSA count (33%)
4	AL, FL, GA, KY, MS, NC, SC, TN	Low income (55%)	Education (54%)	Poverty (53%)
5	IL, IN, MI, MN, OH, WI	pcHPSA count (37%)	Rural county (28%)	Unemployment (25%)
6	AR, LA, NM, OK, TX	Insurance (64%)	Race/ethnicity (54%)	Education (48%)
7	IA, KS, MO, NE	Rural county (52%)	Sparse population (43%)	Older population (37%)
8	CO, MT, ND, SD, UT, WY	Sparse population (80%)	Rural county (71%)	Older population (40%)
9	AZ, CA, HI, NV	pcHPSA count (83%)	Race/ethnicity (59%)	Unemployment (42%)
10	AK, ID, OR, WA	Sparse population (52%)	pcHPSA count (50%)	Unemployment (41%)
All Regions	50 US States + DC	Rurality (36%)	pcHPSA count (30%)	See notes (27%)

Percentages indicate the proportion of pcHPSA counties in each HHS Region or in All Regions that have the particular marker.

Across all 50 U.S. states and the District of Columbia (DC), 27 percent of pcHPSA counties had markers for education, low income, poverty, and sparse population.

As this overview of co-occurring markers shows, certain markers often occurred in combination. Unsurprisingly, for example, 58 percent of pcHPSA counties that had the rurality marker were also characterized by sparse population. Similarly, markers for low income and poverty tended to co-occur, with 76 percent of pcHPSA counties having the poverty marker also having the low income marker.

In addition to examining marker frequencies, we also identified markers associated with the greatest variability across pcHPSA counties in each HHS Region and nationally. These findings help to elucidate individual SDOH factors associated with the greatest degree of disparity among county populations within a single Region (Table 4). For example, in Regions 1, 3, 5, 6, 7, and 8, poverty had the largest variability across county populations in each of these Regions.

**Table 4:** Markers with greatest variability in pcHPSA counties, by HHS Region, 2017.

HHS Region	US States + DC	Variability Source 1	Variability Source 2
1	CT, ME, MA, NH, RI, VT	Poverty	Low income
2	NJ, NY	Education	Sparse population
3	DC, DE, MD, PA, VA, WV	Poverty	Rurality
4	AL, FL, GA, KY, MS, NC, SC, TN	Sparse population	Poverty
5	IL, IN, MI, MN, OH, WI	Poverty	Older population
6	AR, LA, NM, OK, TX	Poverty	Rurality
7	IA, KS, MO, NE	Poverty	Low income
8	CO, MT, ND, SD, UT, WY	Poverty	Race/ethnicity
9	AZ, CA, HI, NV	Unemployment	Poverty
10	AK, ID, OR, WA	Race/ethnicity	Older population
**All Regions**	**50 US States + DC**	**Poverty**	**Low income**

In fact, across HHS Regions, poverty was a dominant source of county-level population variability both nationally and in all HHS Regions except Regions 2 and 10. The sparse population marker was a major source of county-level variability in Region 2 and Region 4, while race/ethnicity was a contributor to variability in Regions 8 and 10, and the older population marker was a contributor to variability in Regions 5 and 10.

## Discussion

### Principal findings

Our findings demonstrate that characteristics measured by markers of medical, economic, and geographic vulnerability were omnipresent in the national population in 2017. Nearly all U.S. county populations had one or more of the characteristics considered here, and, across the 89 percent of U.S. county populations in HRSA-designated pcHPSAs, nearly 90 percent had additional SDOH markers. A small but notable subset of U.S. county populations (206 counties, or 6.6 percent) had 10 or more SDOH markers, and 9 counties (0.3 percent) had the maximum, 13 markers–suggesting higher overall risk levels among residents in those counties in regard to limitations in health service access and poor population health. This latter group, with the most pervasive and complex patterns, lay entirely in HHS Region 4 and 6, roughly the southeastern and south central United States, and appears to reflect the recognized social, economic, and health disparities across Appalachia and in the South. [54, 55] Although, to some extent, each HHS Region had its own pattern of SDOH markers, the most common markers across pcHPSA county populations were rurality (the most common marker nationally), poverty, population sparsity, having a high proportion of older adults, lower education levels, greater racial/ethnic diversity, and higher levels of unemployment.

Many of these markers occurred in combination. Across all U.S. counties, poverty and low income were dominant contributors to the variability of SDOH markers for populations in pcHPSA counties. These findings are particularly important given the fact that poverty is one of the strongest predictors of health, and households in rural, underserved areas tend to experience higher rates of poverty and poorer overall health. [22, 25] Addressing shortages of health professionals in underserved areas is one key part of a broad array of efforts needed to better achieve health equity in these areas.

Several other points deserve emphasis here as well. First, the aim of our analyses is not to validate or critique the pcHPSA process. That process, defined in regulation, reflects health care provider and delivery metrics that are not captured by the makers considered here. This nation-wide shortage of primary care providers is recognized to have variable severity at the sub-national level [6, 31], and the alignment of our markers with this well-recognized mal-distribution of the primary care workforce is an important finding in itself. A measure that correctly identifies and elucidates a widely observed problem affecting most of the country can still be both a valid and a meaningful metric.

Second, the finding that both a high proportion of pcHPSA counties and a high number of counties overall have one or more SDOH markers does not mean that all parts of a particular county are beset by provider shortages or that everyone in a county experiences one or more health vulnerabilities. Rather, these patterns suggest that nearly all U.S. counties have segments of their populations with one or more risk factors, including shortages of primary care providers.

Perhaps more important than the ubiquity of risk factors are the regional differences and complexities in how these markers are distributed and in the attendant regional disparities reflected in those distributions. These disparities are particularly evident in HHS Region 4 and Region 6, roughly covering the southeastern and south central United States, where more than one third of pcHPSA counties in each Region have seven or more of the fifteen SDOH markers considered here. Our analyses help to underscore the population complexities of pcHPSA counties. For example, while many pcHPSA counties across the United States are characterized by elevated rates of poverty, these counties may differ markedly in how poverty is concentrated among children and older adults. For counties that are considered persistent poverty counties by CRS, unique and deeply rooted systemic challenges may present particular constraints. [45] Addressing provider shortages and delivering health care in these areas requires detailed understanding of population characteristics such as those captured by our analyses. [56] There is no “one-size-fits-all” approach to alleviating shortages in areas lacking adequate health provider capacity.

These analyses are not the first to overlay measures of social determinants of health. Indeed, recent and important work by Singh and Lin [57] takes a rigorous look at this issue by examining measures of deprivation across both time and space. What distinguishes our analysis from other recent work is our focus on pcHPSA counties and on how selected risk measures vary across pcHPSA counties, as well as by HHS Region. As discussed below, these analyses may help to inform primary care policy and planning at both federal and non-federal levels.

### Policy and planning implications

Primary care has long been recognized often the first source of care for individuals and families, and as the foundation for maintaining health and wellness across broad health care systems both in the United States and worldwide. [4, 58, 59] Coordinated primary care, delivered through models such as the patient-centered medical home, is seen as a critical piece to improving the patient health care experience and population health, as well as reducing overall health care costs. [60] While the underlying economic and social factors are complex, access to high-quality primary care has been shown to improve longevity, improve quality of life, and help individuals avoid disability, while also reducing rates of emergency department visits and hospitalizations. [60] A recent epidemiological study demonstrated that, over the period of 2005 to 2015, for every 10 additional primary care physicians per 100,000 population, there was an associated increase in life expectancy of 51.5 days. [61] However, during that same period, although the absolute number of primary care physicians across the nation increased, the density of per-population primary care physicians actually decreased due to population increases and disproportionate losses of primary care physicians in some counties (which, in turn, may reflect recruitment/retention challenges in rural areas, urban preferences among some providers, and other factors. [62] This recent study by Basu et al. [61] highlights that access to high-quality primary care is highly uneven across the nation, and that there are notable population health impacts that may result from this access differential.

Access to health care services is a complex, multifactorial problem that is due at least in part to variable “resource” availability–including available sites of care such as hospitals and outpatient practices, and available specialty and primary care providers. [63] Primary care HPSAs are designations that indicate shortages in primary care providers that may be geographic-, population-, or facility-based in nature. [5] HRSA estimates that the population of individuals experiencing a provider shortage in the nation’s 6,433 pcHPSAs is currently around 77 million people–or roughly 24% of the U.S. population–and reflects a shortage of 13,939 primary care providers. [6] Further, HRSA is projecting that, under current patterns of utilization and care delivery, shortages of primary care providers will worsen over the coming years, driven largely by an increasing demand for health care services associated with demographic shifts in the nation’s population together with ongoing challenges in recruiting and retaining primary care providers. [8, 64] This is not solely a problem of primary care workforce magnitude, but also one of geography and distribution. For instance, in 2013, there were approximately 68 primary care physicians per 100,000 residents living in rural areas, which is approximately 19% lower than what the physicians available in urban areas. [64]

A number of other complex factors also limit health care accessibility, including access to primary care services. Such factors include care affordability, physical accessibility, relevance, effectiveness, and acceptability–as well as the presence of cultural barriers and issues of health equity. [36, 63, 65] The term “social determinants of health” aims to capture this collection of factors, which describe the circumstances into which people are born and live, and that are shaped by a wider set of factors such as economics, social policies, and politics. [19] The premise of this investigation was that certain social determinants of health–assessed through defined measures of medical, economic, and geographic vulnerability for which data were readily available–are likely to geographically co-occur with primary care workforce shortages, since access to quality health care, education, housing, transportation, employment, and the system of justice all relate to the societal distribution of resources. These factors, taken all together, in turn impact the prevalence of disease and injury, and overall population health. [19, 20]

Our study shows that nearly all (96 percent) counties in the United States are whole county or partial county pcHPSAs or have one or more risk markers. Perhaps unsurprisingly, this suggests that counties low in certain resources are likely to also be low in other resources. For example, areas with lower average educational attainment levels may also have higher degrees of poverty and unemployment. However, different SDOH markers appear to be variably pervasive in different regions of the country–meaning that the medical, economic, social, and political factors affecting access to primary care, the foundation for maintaining health and wellness across health care systems, are not uniformly contributory. As such, unique subnational factors may need to be considered and addressed while planning and developing public health policies and when making investments in initiatives–including workforce initiatives–intended to drive improvements in primary care health access.

The inherent intertwining of primary care physician shortages with markers of medical, economic, social, and political factors in the nation’s most vulnerable areas makes for great challenges with policy planning and investments, however. Recruitment and retention of primary care providers into rural and other areas with higher degrees of health workforce shortages, evident by lower per-resident numbers of primary care physicians in rural areas, has been a longstanding challenge for the U.S. health care system as well as for the health care systems in other nations, whether they are low- or high-income countries. [63, 66] The largely urban-centralized private medical sector often preferentially attracts health workers over competing rural areas, which also have disadvantages such as weaker labor markets and working and living conditions that may be less desirable to new medical training graduates as potential recruits to such high-need areas. [15, 16, 66] Rural medical practices may have more demanding schedules due to having fewer providers available for service coverage, and potentially due to federal level restrictions regarding reimbursement as well as the state-specific influence of scope of practice limitations for non-physician providers, such as nurse practitioners. [66, 67] Rural practices may also offer lower salaries, which may be an important factor for career choice among new medical school graduates, given the high and rising cost of medical education. [16] In contrast, urban areas are often more attractive locales for health care professionals to practice and live with their families, due to their relative advantages in employment and salary opportunities, social and cultural amenities, higher spousal satisfaction levels and work prospects, and increased access to education opportunities for their children. [56, 67]

Understanding the challenges in addressing provider workforce shortages and geographical imbalances in providers provides a path forward for strengthening health care access and equity in high-need areas. [63] A strategic approach to recruitment and retention of providers into high need areas that is inclusive of multiple interventions seems to be more effective than individual interventions. [63] Strategies can include exposing trainees to health care delivery in high-need areas through rotations or curricular content tailored to the particular needs of the populations served; targeted recruitment of health professionals with ties to and experience with the populations in a specific high-need area; providing personal and professional support; and offering financial incentives that require or entice service in an underserved area in return for educational financial aid. [68, 69]

HRSA is the principal federal agency in the U.S. that is charged with increasing access to effective and efficient basic health care for those who are medically underserved due to barriers in obtaining appropriate and quality care–be those barriers economic, geographic, linguistic, cultural, or attributable to other causes. [70] HRSA administers numerous programs through its $10.7 billion in fiscal year 2020 appropriations that are designed to expand services, increase supplies of health care workers, and improve the distribution of the workforce. For example, the National Health Service Corps supports primary care physicians and other health care professionals who have dedicated themselves to working in underserved, rural, and tribal areas that are located in HPSAs. This program significantly improves the distribution of a much needed primary care health workforce in areas that otherwise would have no access to these health care professionals. Additionally, the Teaching Health Center Graduate Medical Education program supports the resident training of future primary care physicians and dentists in community-based ambulatory care settings in rural and underserved areas. Empirical evidence indicates that physicians trained in these settings are more likely to practice in these same settings, such as health centers, when they complete their training. [69] HRSA’s new Rural Residency Planning and Development program similarly aims to develop new, rural-based residency training programs for physicians in the specialties of family medicine, internal medicine and psychiatry. Lastly, HRSA has awarded cooperative agreement funding to two new Health Workforce Research Centers that will focus their research on issues related to health equity in health workforce education, training, and practice. These centers will strengthen the evidence base for effective education and training programs that incorporate social determinants of health so that the future workforce will be better prepared to ensure health equity for all populations.

In a nationally representative sample of HRSA-funded federally qualified health centers, which disproportionately serve patients whose health is affected by socioeconomic disadvantages, patients reported a particularly high prevalence of social risk factors–including unstable housing and employment, lower education levels, and poverty. [71] HRSA is investing $5.6 billion total in health center program funding in FY2020 in order to support the nearly 1,400 health centers that provide care to approximately 27.2 million underserved patients. [70] HRSA’s primary care workforce programs, together with an understanding of population risk factors and disparities, are essential for addressing the nation’s mal-distribution of primary care providers and in particular ensuring sufficient staffing at health centers in medically underserved areas. [72]

### Strengths and limitations

Our analyses offer a national view of the distribution of vulnerable populations in pcHPSAs across U.S. states and in the District of Columbia. By selecting a range of SDOH measures, we highlight not only the ubiquity but also the complicated needs of populations who may be geographically, economically, or medically vulnerable. Moreover, by overlaying SDOH measures with pcHPSA counties, our analyses provide important context for the consideration of policy implications specific to primary care and related workforce programs, including HRSA’s programs.

As with any analysis, there are limitations. First, it must be emphasized that HRSA’s designated pcHPSAs are based in regulation and so may not fully capture all primary care provider shortages at a particular point in time. While pcHPSAs offer a useful lens for our national analyses, we caution that more granular analyses at state and sub-state levels may require greater detail in identifying provider shortages than that afforded by looking at pcHPSAs.

We also did not attempt to estimate the number of people affected by either individual or collective vulnerabilities. While our use of proportions and categorical variables permitted us to evaluate characteristics across U.S. counties using a consistent suite of scales, our analyses cannot fully measure problem magnitude with respect to the size of vulnerable populations. Particularly in densely populated areas, more detailed analyses, to include estimates of population size, will be important in guiding health services planning at state and sub-state levels. In fact, replicating our analyses at the state and county levels may be beneficial to inform policy-makers about local decisions and to help find efficiencies in resource allocation and inter-related health policies.

Another limitation, commonly termed the ecological fallacy, involves assigning a single estimate or attributing a particular characteristic to every individual in a population. This is a well-recognized limitation of aggregated data analysis [73–75], and considerable caution is needed when inferring information about individuals from group-level measures. For example, it cannot be assumed that, in a county identified as a persistent poverty county, all individuals in the county experience persistent poverty to the same extent. However, our focus here is not on individuals, nor, as noted above, is it on population size. Rather, our aim is to elucidate regional complexities. Indeed, an important element of our analyses involves description of these regional or system-level patterns. As Galea and colleagues have recently discussed [76–78], understanding system level effects is essential to sound planning and policy development. In addition, capturing the complex effects of social determinants of health holistically is analytically challenging, and reliable data sources for some determinants are limited, particularly at the county level. Measures that accurately and completely capture the essence of certain determinants of health equity, such as social justice, do not exist.

Similarly, as a number of researchers have discussed, assessing rurality for the purposes of health care analyses is a complex undertaking [17, 79], and requires consideration of population size, population density, proximity to health care and other services, local and regional infrastructure, and other factors. While our blending of the RUCC and UIC classifications attempts to capture some of this complexity, it can reflect only a portion of the full nuance. Although beyond the scope of our analyses, we are intrigued by recent research from Doogan et al. [80] that proposes a “geographic isolation scale” for examining rural health disparities, and we expect to incorporate more rigorous analyses of rurality in future work.

We would also note the cross-sectional nature of our analyses. They are snapshots in time and, with the exception of the CRS persistent poverty indicator, our SDOH markers do not account for trends over time in population characteristics. Such trend analyses are indeed important, and represent an important next step in refining this work.

Finally, we acknowledge our use of “convenience” measures available in AHRF, rather than conducting primary data collection ourselves or retrieving data directly from the Census Bureau, ERS, and other resources. However, AHRF provides a comprehensive, county-level picture, and affords an opportunity to efficiently examine multiple markers at a common point in time and with consistent data validation checks applied. Again, an important next step will be to augment these analyses with additional variables directly from the U.S. Census Bureau and other primary data sources, as well as possible primary data collection. However, for the purposes of providing an initial county-level view, the AHRF affords an opportunity to examine pcHPSA counties across a range of medical, economic, and geographic measures.

### Conclusion

Nationally, 89 percent of U.S. counties are classified as either whole county or partial county pcHPSAs and 96 percent of U.S. counties have one or more population risk markers, suggesting that medically, economically, and geographical vulnerable groups are nearly ubiquitous throughout the nation. Across all U.S. pcHPSA counties, the most common markers are rurality, having multiple pcHPSAs in a single county, education, low income, poverty, and sparse population. pcHPSA populations in HHS Region 4 and Region 6 have the most pervasive and complex risk patterns, and reflect the recognized social, economic, and health disparities across Appalachia and in the South. Collectively, these ecological measures of county-level population characteristics may help inform health workforce and health care planning at all levels, and, by shining a light on the risk complexities affecting U.S. populations, our findings may also help to guide efforts to strengthen the delivery of primary care in areas characterized by provider shortages.

## Supporting information

S1 File (DOCX)
