# Supplementary material for: The geographic alignment of primary care Health Professional Shortage Areas with markers for social determinants of health
Source: PLoS One. 2020 Apr 24;15(4):e0231443. doi: 10.1371/journal.pone.0231443 (PMC7182224; doi:10.1371/journal.pone.0231443)
Supplement: S1 File — (DOCX) [file pone.0231443.s001.docx]

**Supplemental Material**
